# Mind–body effects of mindfulness-based training in athletes: a preliminary randomized controlled trial

**DOI:** 10.3389/fpsyg.2026.1755043

**Published:** 2026-02-19

**Authors:** Carmelo Campo, Filippo Cellucci, Giacomo Treggiari, Giorgio Alfredo Spedicato, Stefano Lasaponara, David Conversi

**Affiliations:** 1 Department of Psychology, Sapienza University, Rome, Italy; 2 Department of Experimental Medicine, Sapienza University, Rome, Italy; 3 Department of Statistics and Quantitative Methods, Milano-Bicocca University, Milan, Italy; 4 Neuropsychology of Attention Lab, IRCCS Fondazione Santa Lucia, Rome, Italy; 5 Research Center in Neurobiology “Daniel Bovet”, Rome, Italy

**Keywords:** athletes, cognitive inhibition, HRV, interoceptive awareness, mindfulness, muscle flexibility, pulmonary function

## Abstract

**Introduction:**

Previous research has shown that mindfulness practice is associated with a range of indicators related to better mental and cognitive health. Emerging evidence also supports benefits for physical health, although these findings are not yet comprehensive. Consequently, this practice is attracting increasing interest in sports, despite the current lack of extensive research and limited application in this context.

**Methods:**

This study analyzed the effects of an 8-week mindfulness-based training in athletes on several psychological and physiological variables that may influence sports performance. Forty-six athletes were recruited and randomly assigned to a study group, which joined the mindfulness-based training, or a waitlist control group. Levels of interoceptive awareness, cognitive inhibition, heart rate variability (HRV), pulmonary function, and muscle flexibility were assessed before (T0) and after (T1) the treatment.

**Results:**

Results showed statistically significant improvements in interoceptive awareness and SDNN parameter of HRV in the study group compared to the control group following the treatment.

**Discussion:**

These preliminary results partially support the holistic health benefits of mindfulness and encourage the integration of mindfulness techniques into sports training. Future research should explore these findings in larger samples and for longer treatment durations.

## 1 Introduction

In high-level sports context, it is recognized that optimal performance depends not only on athletes’ physical capabilities, but also on their mental abilities and psychophysiological conditions, such as wellbeing and stress (Wang et al., 2025; Schinke et al., 2018). Consequently, sport psychology is increasingly investigated in sport science, although the integration of psychological interventions into athletic training programs remains scarcely applied (Wang et al., 2025). A psychological technique that is attracting growing interest for its effectiveness—even within the sports field—is mindfulness. It is described as “*the awareness that emerges through paying attention on purpose, in the present moment and non-judgmentally, to the unfolding of experience*” (Kabat-Zinn, 2003). Existing literature indicates that mindfulness-based training can generate positive psychological effects, including improved wellbeing, better emotional and behavioral regulation, and reduced stress (Keng et al., 2011; Spinelli et al., 2019; Accoto et al., 2021). As a result, it is starting to be applied in the sports field, with the aim of promoting athletes’ health and performance (Gardner and Moore, 2017; Gledhill, 2021). Several sport-specific mindfulness protocols have been developed to adapt traditional techniques to athletes’ demands, such as Mindfulness Meditation Training for Sport (MMTS) (Baltzell et al., 2014) or Mindful Sport Performance Enhancement (MSPE) (Kaufman et al., 2009). Consistent with this trend, accumulating evidence indicates that mindfulness-based interventions in athletes are associated with beneficial psychological outcomes—such as enhanced sport-related mental skills—as well as measurable improvements in sport performance (Si et al., 2024; Wang et al., 2023; Sappington and Longshore, 2015; Josefsson et al., 2019; Bühlmayer et al., 2017). However, the underlying psychophysical mechanisms through which mindfulness influences athletic performance remain not yet fully understood.

Mindfulness has been conceptualized as a metacognitive practice involving higher cognitive abilities to observe and regulate ongoing mental processes, through intentional allocation of attentional resources (Jankowski and Holas, 2014; Dahl et al., 2015; Lutz et al., 2008; Kabat-Zinn, 2003). During practice, two main attentional techniques are typically adopted: focused attention (FA), directing attention to an object (e.g., breath, body sensations) and, when the mind inevitably wanders, redirecting attention; open monitoring (OM)—which is adopted in advanced stages—directing attention to the overall present experience while maintaining vigilant and non-reactive monitoring of the consciousness flow (Dahl et al., 2015; Lutz et al., 2008). FA has been linked to selective attention mediated by fronto-parietal networks, whereas OM to sustained attention mediated by the noradrenergic system, both coordinated by executive functions engaging the anterior cingulate cortex and dorsolateral prefrontal cortex (Chiesa et al., 2011; Petersen and Posner, 2012). Accordingly, evidence indicates that mindfulness-based training leads to improvements in various attention-measuring tasks (Sumantry and Stewart, 2021; Verhaeghen, 2021; Chiesa et al., 2011)—particularly those targeting executive processes such as working memory and cognitive inhibition (Sumantry and Stewart, 2021; Verhaeghen, 2021; Cásedas et al., 2020)—and induces consistent brain changes in attentional networks (Tang et al., 2015).

Emerging evidence suggests that mindfulness could also have positive effects on physical health (Creswell et al., 2019; Grossman et al., 2004; Pascoe et al., 2017), although these findings are not yet comprehensive. The strongest evidence concerns cardiovascular health (Scott-Sheldon et al., 2020), chronic pain (Hilton et al., 2017), and inflammatory and immunological markers (Black and Slavich, 2016; Heckenberg et al., 2018; Pascoe et al., 2017; Rosenkranz et al., 2013; Higgins et al., 2022). The mechanisms underlying these changes primarily involve alterations in brain activity related to stress, emotions, and interoception, including increased connectivity in prefrontal, cingulate, and insular regions and reduced reactivity in limbic areas (Creswell et al., 2019; Tang et al., 2015). These neural changes can then affect the body through the hypothalamic–pituitary–adrenal axis (HPA) and the autonomic nervous system (ANS) (Creswell et al., 2019; Pascoe et al., 2017). In response to stress or anxious stimuli, the HPA axis release powerful molecules in the bloodstream—such as cortisol—and its chronic activation can lead to immune suppression, increased inflammation, and accelerated biological aging (Charmandari et al., 2005). In this regard, mindfulness has been shown to reduce cortisol secretion (Pascoe et al., 2017; Heckenberg et al., 2018). On the other hand, the ANS comprises fibers originating from the brainstem and spinal cord and regulates autonomic functions through two opposing subsystems: the sympathetic nervous system, which is activated in stressful or dangerous situations and increases heart and respiratory rates, as well as muscle response; and the parasympathetic nervous system, which is activated in conditions of rest and safety and decreases these processes (Gibbons, 2019). Notably, sympathetic hyperactivation and parasympathetic hypoactivation have been associated with prolonged stress conditions (Gibbons, 2019). Conversely, mindfulness has been shown to promote ANS balance, as reflected by better heart rate variability (HRV) (Tung and Hsieh, 2019; Heckenberg et al., 2018). HRV is a non-invasive method for observing the heart’s ability to adapt to impulses from the ANS (Sztajzel, 2004), representing a valid measure of ANS balance, stress adaptability, and mental and physical health (Kemp and Quintana, 2013).

As mentioned, ANS activity is also linked to both respiratory rate and muscle tone. For example, sympathetic hyperactivation—and the associated states of stress and anxiety—has been associated with rapid and irregular breathing rates (Jerath et al., 2015) and muscle tension (Pluess et al., 2009). Regarding the relationship between mindfulness and respiration—beyond the above-mentioned autonomic regulation that could benefit this process—the ability to maintain awareness of breathing is particularly emphasized in this practice, through techniques that include attention to breath sensations and respiratory muscle movements (Kabat-Zinn, 2003; Jerath et al., 2015). In this regard, mindfulness has been linked with reductions of respiratory rate in healthy meditators (Karunarathne et al., 2024; Wielgosz et al., 2016), and with benefits for individuals with pulmonary disorders—both in terms of psychological outcomes (Arefnasab et al., 2013; Paudyal et al., 2018; Li et al., 2023) and airway inflammation biomarkers (Higgins et al., 2022). However, no other evidence has been found yet about effects on pulmonary function parameters (Karunarathne et al., 2024; Arefnasab et al., 2013; Paudyal et al., 2018). Similarly, although mindfulness has shown to reduces stress, anxiety and sympathetic hyperactivation (Keng et al., 2011; Tung and Hsieh, 2019; Heckenberg et al., 2018)—factors that can increase muscle tension (Pluess et al., 2009)—and although it is theoretically assumed to promote bodily relaxation (Kabat-Zinn, 2003; Luberto et al., 2020), evidence on its direct effects on muscle parameters remains minimal. In addition to studies reporting improvements following mindfulness in musculoskeletal disorder symptoms (Hilton et al., 2017; Cruze and Games, 2021), there is evidence reporting a decrease in the electrical activity of muscle fibers measured through electromyography (Crescentini et al., 2016).

In light of all these assumptions, and returning to the sports context, it is therefore conceivable that psychophysiological stress and the related autonomic balance could influence athletic performance. For example, the maladaptive muscle tension that occurs during chronic stress response can reduce range of motion, flexibility, and motor coordination (Williams and Andersen, 1998; Olmedilla-Zafra et al., 2017). These patterns could increase injury risk (Williams and Andersen, 1998; Olmedilla-Zafra et al., 2017; Witvrouw et al., 2003) and impair sports performance (Olmedilla-Zafra et al., 2017; Tossici et al., 2024; Skopal et al., 2024). The cardiovascular and pulmonary functions are also critical for performance—especially in endurance activities—by supplying adequate blood and oxygen to muscles, improving the ability to sustain prolonged exertion and recover quicker (Mazaheri et al., 2021). Pulmonary function further support peak oxygen uptake and ventilatory efficiency, as well as exercise capacity in most sports (McNeill et al., 2022). Moreover, attention to external stimuli and its executive components are influential in competitive sports performance, enabling athletes to ignore distractions, maintain focus on the main activity, and flexibly adapt to constantly changing game strategies (Brimmell et al., 2024; Moran, 2012). At the same time, attention to internal stimuli, also known as interoception, enables athletes to better motor coordination or to adequately manage training intensity based on physiological signals, such as those of fatigue, thus reducing the risk of over-exertion-related injuries (Li et al., 2021; Bruscolotti et al., 2020; Gledhill, 2021). Given the relevance of these variables for sport performance, it is important to better understand whether interventions such as mindfulness can promote adaptive changes in these domains within athletic populations. Therefore, the purpose of the current study was to explore the effects of mindfulness-based training in athletes on several psychological and physiological variables, such as interoceptive awareness, cognitive inhibition, heart rate variability, pulmonary function, and muscle flexibility. We hypothesized that a study group who joined an 8-week mindfulness-based training would show significant improvements in these variables in these variables following the intervention compared to a control group.

## 2 Methods

### 2.1 Participants

As a first recruitment step, three different adjacent sports centers near Rome (Italy) were contacted. They were asked to offer an additional service to their members that included lifestyle improvement courses and medical surveys, provided free of charge and within the framework of a research project. Only one sports center became available and joined the project. The second step of recruitment was conducted with the support of staff members of the participating sports center, who informed potential participants about the study and facilitated contact with the research team. There were no cash incentives either for staff members or for the participants. As inclusion criteria, we only accepted participants without any experience in meditation and who practiced amateur sports from two to four times a week. Initially, 54 subjects were recruited, but 8 of them were later excluded due to withdrawal from the study or incomplete data collection. Therefore, a total of 46 individuals, aged between 18 and 59, participated in the study. Most participants practiced CrossFit (*n* = 30), while the remaining subjects practiced heterogeneous sports disciplines such as Weightlifting (*n* = 8), Martial arts (*n* = 4), Football (*n* = 2), and Rugby (*n* = 2). The candidates were equally and randomly divided into two examination groups, except that an attempt was made to ensure that the distribution was homogeneous regarding gender, age, and sport typology. Thus, the total of the study group was 23 participants (16 males and 7 females, with an average age of 34.52 ± 9.61), and the control group was also 23 participants (15 males and 8 females, with an average age of 33.65 ± 9.98). This sample size seems to be comparable to that used by many previous studies investigating similar phenomena with similar experimental design (Keng et al., 2011; Grossman et al., 2004; Scott-Sheldon et al., 2020; Heckenberg et al., 2018; Bühlmayer et al., 2017). Moreover, a post-hoc sensitivity power analysis was performed to estimate the minimum detectable effect size based on the final sample size. Assuming a level of *α* = 0.05 and a statistical power of 1 − *β* = 0.80, the analysis indicated that the available sample of 46 participants was sufficient to detect a minimum effect size of Cohen’s *f* = 0.42, which reflects a medium-to-large effect according to conventional thresholds (Cohen, 2013).

### 2.2 Measures

#### 2.2.1 Interoceptive awareness

Interoceptive awareness was assessed through the MAIA questionnaire (Multidimensional Assessment of Interoceptive Awareness) (Mehling et al., 2012). It is a self-report assessment tool used to measure interoceptive or somatic awareness, representing the ability to perceive, access, and integrate signals from within the body in everyday life. The questionnaire consists of 32 items, five of which are reverse-scored, divided into 8 subscales: Noticing, Not-Distracting, Not-Worrying, Attention Regulation, Emotional Awareness, Self-Regulation, Body Listening, and Trust. An example is “*When I am tense, I notice where the tension is located in my body*”. Participants rate items using a six-point Likert scale ranging from “0 = Never” to 5 = Always”. We used the Italian version of MAIA (Calì et al., 2015), which has shown to have acceptable psychometric results that support the construct validity, the internal reliability, and the factorial structure of the original questionnaire. In the present study, a single composite score was calculated, representing the total mean obtained by summing all item scores and dividing by 32. The internal consistency was evaluated using both Cronbach’s *α* (Nunnally, 1978) and McDonald’s *ω* (McDonald, 1999) coefficients in the total sample. The scale showed excellent reliability, with *α* = 0.95 and *ω* = 0.96 at T0, and *α* = 0.94 and *ω* = 0.94 at T1, indicating adequate internal consistency in this population of athletes.

#### 2.2.2 Cognitive inhibition

Cognitive inhibition was assessed using the Stroop task (MacLeod, 1991). This test is primarily used to obtain information about executive and attentive functioning (Scarpina and Tagini, 2017). The test was implemented using the “*Inquisit 6*” software from the “*Millisecond*” platform, which provides a library of psychological tests in virtual version (Barzykowski et al., 2021). The version used was “*Color-Word Stroop with Keyboard Responding*”. The test was administered through a 16″ portable computer (Lenovo IdeaPad), placed in front of the seated participant, and isolating headphones to ensure a distraction-free environment. The task involved 84 trials presented in a random order. Each trial began with a central fixation cross of 500 ms, followed by the stimulus until response (max 2,000 ms). A blank inter-trial interval of 1,000 ms separated successive trials. The stimuli consisted of words related to four colors (blue, yellow, green, red) and rectangles displayed in the same four colors. The trials were equally divided into three conditions: congruent, in which word meaning matched with the font color; “*incongruent*,” in which word meaning did not match with the font color; and “*control*,” in which only colored rectangles appeared. Participants were asked to respond as quickly and accurately as possible via four keyboard keys (Z, X, N, M) according to the four distinct colors in which the stimuli were displayed. Following a brief practice phase, participants started the test, which lasted approximately 3 min. The accuracy and average rt (response time) were registered for each condition. One participant did not perform the task due to color blindness, while three participants were excluded from the analyses because their accuracy was below 60% in at least one condition (Hedge et al., 2018). Cognitive inhibition was then evaluated using the Interference index (Scarpina and Tagini, 2017), which was computed as follows:


$$I=RT_{\text{correct incongruent}}-\frac{\left(RT_{\text{correct congruent}}+RT_{\text{correct control}}\right)}{2}$$

According to this, a lower score in Interference value indicates better inhibitory control.

#### 2.2.3 Heart rate variability

HRV was computed from R–R intervals recorded through a “*Polar H10*” monitor (sampling rate: 1,000 Hz), chosen for its validity and reliability in acquiring HRV data (Schaffarczyk et al., 2022). Before the recording, participants were seated in a chair while the sensor was moistened with a propylene glycol gel to increase its conductivity and then positioned centrally on the participant’s chest, just below the pectoral muscles, ensuring direct skin contact through an elastic strap (Schaffarczyk et al., 2022). After that, participants were given 3 min to rest and breathe normally. R–R intervals were recorded during the final minute of this period, corresponding to a one-minute ultra-short-term recording (Baek et al., 2015; Shaffer et al., 2016). The data were collected through the mobile application “*Elite HRV*” (Moya-Ramon et al., 2022), connected to the monitor via Bluetooth. These data were then processed through the software “*Kubios HRV*” (Tarvainen et al., 2014), which allows in-depth analysis of HRV parameters. It detects and corrects artifacts and noise in the R–R interval series before calculating HRV through algorithms to ensure a more accurate and valid measurement (Alcantara et al., 2020). In the present study, the medium correction level was applied. The extracted HRV parameters were SDNN (Standard Deviation of Normal to Normal), index of general autonomic functioning, and RMSSD (Root Mean Square of the Successive Differences), index of parasympathetic functioning (Shaffer and Ginsberg, 2017).

#### 2.2.4 Pulmonary function

Pulmonary function was assessed using the Spirometry test. This test is used to assess several aspects of pulmonary efficiency such as flexibility and volumetric capacity of the lungs, obstruction, resistance, or inflammation in the airways (Graham et al., 2019). Data collection of spirometry was conducted using the “*MIR Spiro*” portable spirometer (Medical International Research, Rome, Italy) (Degryse et al., 2012) and following the standard methods of use proposed in the scientific literature (Graham et al., 2019). Participants were instructed to remain seated and wear a disposable mouthpiece with a nose clip to prevent air leakage. After a brief familiarization, they performed at least three maximal expiratory maneuvers, consisting of a deep inhalation to total lung capacity followed by a rapid and forceful exhalation into the mouthpiece until no more air could be expelled. The maneuver with the highest acceptable values was selected for analysis. One participant did not perform the test due to acute upper respiratory symptoms at the time of the first assessment. The spirometer was connected via Bluetooth with the “*Mir Spiro*” software for data acquisition and processing. The extracted parameters of interest were: FEV1 (Forced Expiratory Volume), which indicates the volume of air exhaled during the first second of a forced exhalation after a maximal inhalation; FVC (Forced Vital Capacity), which is the volume of total air expelled in a forced exhalation and after a maximal inhalation; FEV1/FVC (Tiffeneau Index), which is the ratio of the first two indices and reflects the degree of airway obstruction or restriction (Graham et al., 2019).

#### 2.2.5 Muscle flexibility

Muscle flexibility was assessed through the Sit and reach test. This test is primarily used to measure the flexibility of the posterior kinetic chain, specifically focusing on the hamstrings and lumbar extensors (Mayorga-Vega et al., 2014). We utilized the standard version of this test (Lemmink et al., 2003), which has shown greater criterion-related validity than other modified versions (Mayorga-Vega et al., 2014). The instrument used was a specially constructed cube-shaped box with a slide ruler attached to the top. The height of the box was about 30 cm, while the measuring tape on top started at 0 cm and was graduated in 0.5 cm intervals. The procedure required the participant to sit on the floor with the legs stretched forward and the soles of the bare feet placed flat against the box. Then, the participant had to extend the upper body forward to reach the slide with the hands and push it as far away as possible, holding the position for 2 s. The score was the shift of the slide in cm, with higher scores indicated better muscle flexibility. Three measurements were recorded for each participant, and the best value of these three was used for the analysis (Lemmink et al., 2003).

### 2.3 Intervention

The mindfulness-based training was conducted by a qualified mindfulness instructor with an international AIOC certificate (Academy of Certified Instructors and Operators). It was developed inside a room of the same gym, specially equipped to guarantee a quiet and relaxing environment. The room was softly lit, isolated from external noise, at a constant temperature of 22–24 °C, and had a soft and elastic floor, known as “*tatami*,” allowing participants to meditate in a sitting or lying position. The mindfulness lessons lasted 8 weeks, with a frequency of 2 times a week, for a total of 16 lessons. Each lesson included 15 min of introductory explanations about the practice and 45 min of guided mindfulness, for a total of 60 min of lesson. The content of each lesson varied from one session to the next, including different mindfulness exercises based on two of the main mindfulness techniques in the literature, namely “*focused attention*” (FA) and “*open monitoring*” (OM) (Dahl et al., 2015; Lutz et al., 2008). In the first case, participants were guided to relax and focus their attention on an object, such as breath, mental images, parts of the body, and sensations in the present moment. In the second case, the participants were instructed to monitor everything that emerged from the experience, maintaining an acceptance, non-judgment, and non-reaction attitude. During the first half of the lessons, the FA technique was primarily used, while in the second half the OM technique became the main technique. This approach was developed according to scientific literature, which suggests that FA is typically utilized in the initial stages of mindfulness training, while OM is generally introduced in more advanced stages (Dahl et al., 2015; Lutz et al., 2008). In addition, we encouraged the study group to follow home-based supplementary training. For this purpose, guided meditations recorded by the same instructor were provided to participants through an online platform called “*Notion*.” At the end of the course, the average mindfulness time per person, including both on-site sessions and home-based practice, was 528 ± 151 min. Regarding the control group, participants were placed on a waiting list for the mindfulness-based training, as previously done in literature (Spinelli et al., 2019). During the waiting period, participants were instructed to continue their regular sports routines and to not engage in any mindfulness practices outside of the study.

### 2.4 Procedure

Firstly, potential participants were contacted to schedule a slot during the data collection week in the same gym. The available candidates were assigned an identification number and were then randomized into the two groups. After their arrival at the appointment, individuals assigned to the study group were given an introduction to the course structure and a schedule of the upcoming meetings, while individuals assigned to the control group were given instructions about the waiting period. Participants performed the tests in a quiet room of the gym in the following order: HRV, Stroop test, Spirometry and Sit and reach. Physiological tests were carried out by the research team in collaboration with a thoracic surgeon and pulmonologist from outside the research group. These tests were conducted before the start of physical training to avoid possible distortions of the results due to fatigue or physical stress, taking care to warn the candidates to rest adequately, wear comfortable and flexible clothing, and avoid the consumption of heavy foods, alcohol, caffeine, nicotine, and drugs (Ferguson, 2014). Moreover, for each participant, these assessments were collected within the same half of the day (i.e., morning or afternoon) at T0 and T1, to minimize potential within-subject circadian confounding factors, especially in light of HRV measurement variability (Damoun et al., 2024). The self-report questionnaire was administered through the online platform (i.e., *Notion*). At T0, it included questions about the baseline characteristics, while at T1 it comprised a section for the mindfulness group to report the amount of practice at home. The attendance of participants to the mindfulness lessons was tracked by the research team session-by-session. The initial tests (T0) were made the week before the start of the mindfulness lessons, while the final tests (T1) were made in the week immediately following the end of the meetings. The privacy rights of human subjects have been observed, and each participant compiled and signed an informed consent suitable for processing personal data and administering the tests (R.E 2016/679). This study was conducted in accordance with the principles outlined in the Declaration of Helsinki. Ethical approval was granted by the Ethics Committee of the Department of Psychology of “La Sapienza” University of Rome.

### 2.5 Statistical analysis

We collected a set of outcomes at two separate times, pre-treatment (T0) and post-treatment (T1). The aim of the study was to determine if the treatment significantly impacted the dynamics of these outcomes. For this purpose, we employed a repeated measures linear mixed effects modelling framework (Bates et al., 2015). For each outcome, the model included: time (within-subject factor), group (between-subjects factor), their interaction (time × group), and age and gender as covariates. A random intercept for each participant was included to account for subject-level heterogeneity and the correlated nature of repeated measurements. Statistical significance was determined using a *p*-value threshold of *α* = 0.05. Finding a statistically significant time × group interaction effect would suggest that the treatment substantially affects the direction of the outcome. For outcomes with a statistically significant interaction effect, we conducted a more in-depth post-hoc analysis using estimated marginal means (Lenth, 2023). For all fixed effects in the mixed models, approximate effect sizes were derived from the *F*-statistics (Lenth, 2023). Specifically, we computed Cohen’s *f*^2^ as:


$$f^{2}=\frac{F\cdot {\mathrm{df}}_{1}}{{\mathrm{df}}_{2}}$$

and its square root:


$$f=\sqrt{f^{2}}$$

These quantities provide interpretable measures of the magnitude of each model term, including the critical time × group interaction. All analyses were conducted in R (R Core Team, 2023), using packages from the tidyverse ecosystem (Wickham et al., 2019) and gtsummary for descriptive summaries (Sjoberg et al., 2021).

## 3 Results

Descriptive statistics for baseline data of mindfulness and control groups are shown in Table 1. Results from Welch Two Sample t-test showed no statistically significant differences between the two groups in continuous variables such as age, BMI, and sport time per week (Table 1). Results from Pearson’s Chi-squared test showed no statistically significant differences between the two groups in categorical variables such as Gender, Habitual smoker, Musculoskeletal disorders, Metabolic and/or autoinflammatory disorders, and Lung or respiratory disorders (Table 1). Descriptive statistics for the outcomes of mindfulness and control groups at T0 and T1 are shown in Table 2, together with the p-values for the time × group interaction from the linear mixed-effects models. All parameter estimates of linear mixed-effects models are reported in Supplementary Table S1.

**Table 1:** Baseline data of mindfulness and control groups. Continuous variables are reported as mean (stand. dev.), with *p*-values from Welch’s two-sample t-test. Categorical variables are reported as counts, with *p*-values from Pearson’s chi-squared test.

Variable	Control, *N* = 23	Mindfulness, *N* = 23	*p*-value
Age	33.65 (9.98)	34.52 (9.61)	0.765
Gender (female/male)	8/15	7/16	0.753
BMI	24.72 (3.10)	23.30 (2.08)	0.076
Sport time per week (min)	344 (140)	356 (147)	0.783
Habitual smoker (no/yes)	14/9	16/7	0.536
Musculoskeletal disorders (no/yes)	17/6	18/5	0.730
Metabolic and/or autoinflammatory disorders (no/yes)	23/0	21/2	0.148
Lung or respiratory disorders (no/yes)	18/5	21/2	0.218

**Table 2:** Mean (stand. dev.) for outcomes of mindfulness and control groups at T0 and T1, and *p*-value of time × group interaction from linear mixed-effects models for each outcome.

Variable	Measure	Control (*N* = 23)		Mindfulness (*N* = 23)		*p*-value
		T0	T1	T0	T1	
Interoceptive awareness	MAIA	2.82 (0.75)	2.85 (0.62)	2.69 (0.77)	3.09 (0.67)	0.023
Cognitive inhibition	Interference	312.64 (383.04)	164.32 (214.23)	327.65 (235.40)	299.24 (183.71)	0.176
HRV	SDNN (ms)	63.27 (35.01)	60.55 (35.74)	61.47 (28.26)	79.18 (33.86)	0.002
	RMSSD (ms)	53.09 (33.50)	54.17 (42.23)	55.87 (31.39)	68.35 (37.43)	0.149
Pulmonary function	FEV1 (L)	4.24 (0.91)	4.30 (1.01)	3.97 (0.90)	4.04 (0.95)	0.849
	FVC (L)	5.17 (1.10)	5.20 (1.23)	4.76 (1.00)	4.75 (1.09)	0.575
	FEV1/FVC (%)	82.17 (6.12)	82.83 (5.93)	83.23 (5.42)	85.13 (4.83)	0.289
Muscle flexibility	Sit and reach (cm)	8.96 (8.83)	8.55 (8.87)	6.97 (8.21)	7.41 (7.98)	0.151

### 3.1 Interoceptive awareness

**Figure 1:**
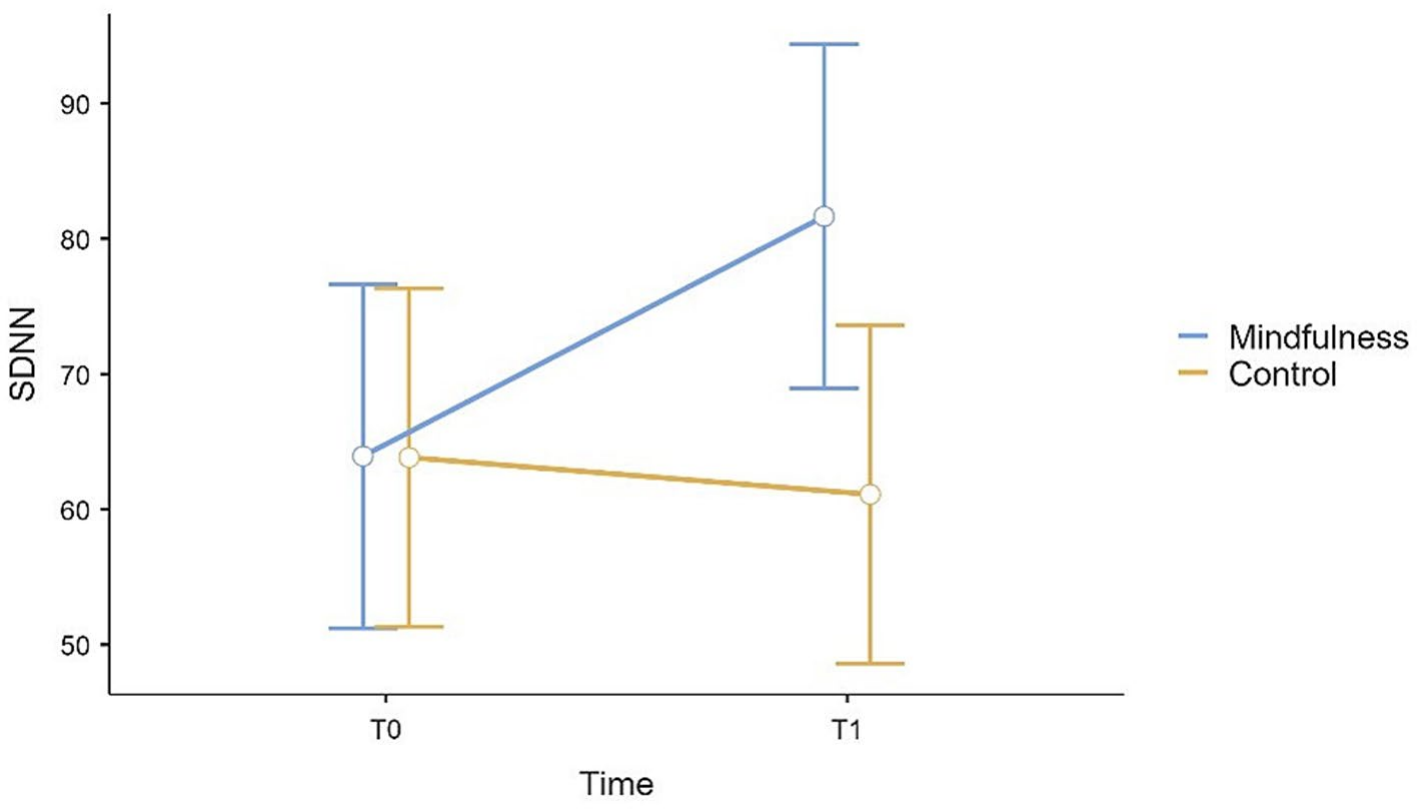
Plot (mean and 95% CI error bars) of variations from T0 to T1 in MAIA scores for mindfulness and control groups.

The analysis revealed a statistically significant main effect of time in MAIA scores, *F*(1, 44) = 7.79, *p* = 0.008, *f* = 0.42, whereas the main effect of group was not statistically significant, *F*(1, 42) = 0.05, *p* = 0.830, *f* = 0.03. A statistically significant time × group interaction was observed, *F*(1, 44) = 5.56, *p* = 0.023, *f* = 0.36, indicating that changes over time differed between groups. Among the covariates, age was not statistically significant, *F*(1, 42) = 3.63, *p* = 0.063, *f* = 0.29, as well as gender, *F*(1, 42) = 0.23, *p* = 0.632, *f* = 0.07. Post-hoc analyses showed a statistically significant increase in MAIA scores from T0 to T1 in the study group (*E* = 0.40, SE = 0.11, 95% CI [0.18, 0.62], *t* = 3.64, *p* < 0.001), whereas no statistically significant change was observed in the control group (*E* = 0.03, SE = 0.11, 95% CI [−0.19, 0.25], *t* = 0.31, *p* = 0.761) (Figure 1). All pairwise post-hoc contrasts for MAIA are reported in Supplementary Table S2.

### 3.2 Cognitive inhibition

The analysis revealed a statistically significant main effect of time in Interference scores, *F*(1, 40) = 4.12, *p* = 0.049, *f* = 0.32, whereas the main effect of group was not statistically significant, *F*(1, 38) = 1.01, *p* = 0.322, *f* = 0.16. No statistically significant time × group interaction was observed, *F*(1, 40) = 1.89, *p* = 0.176, *f* = 0.21, indicating that changes over time did not significantly differ between groups. Among the covariates, age was not statistically significant, *F*(1, 38) = 2.18, *p* = 0.148, *f* = 0.23, as well as gender, *F*(1, 38) = 3.50, *p* = 0.069, *f* = 0.30.

### 3.3 Heart rate variability

**Figure 2:**
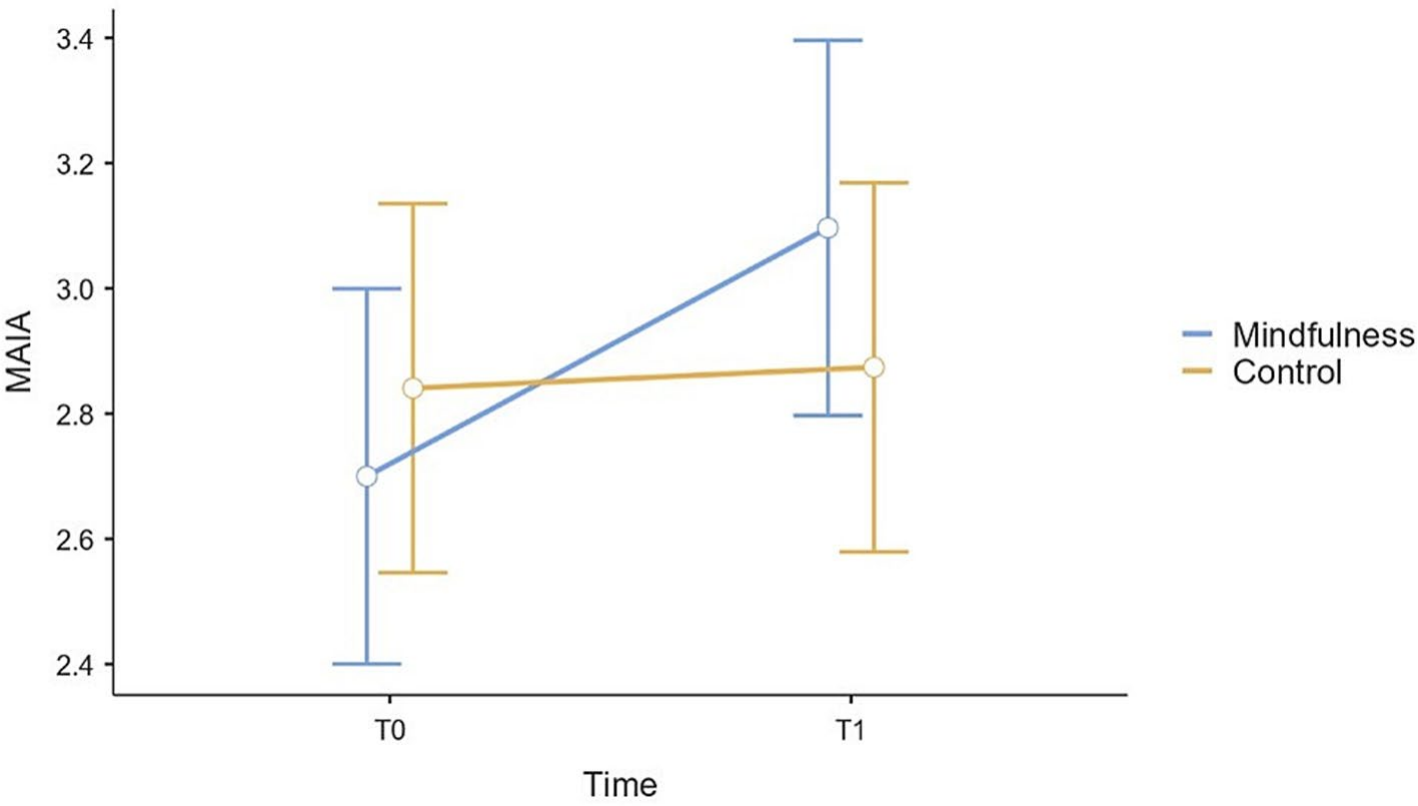
Plot (mean and 95% CI error bars) of variations from T0 to T1 in SDNN scores for mindfulness and control groups.

The analysis revealed a statistically significant main effect of time in SDNN scores, *F*(1, 44) = 5.69, *p* = 0.021, *f* = 0.35, whereas the main effect of group was not statistically significant, *F*(1, 42) = 1.64, *p* = 0.207, *f* = 0.20. A statistically significant time × group interaction was observed, *F*(1, 44) = 10.59, *p* = 0.002, *f* = 0.49, indicating that changes over time differed between groups. Among the covariates, age was statistically significant, *F*(1, 42) = 17.11, *p* < 0.001, *f* = 0.64, while gender was not statistically significant, *F*(1, 42) = 1.00, *p* = 0.322, *f* = 0.15. Post-hoc analyses showed a statistically significant increase in SDNN from T0 to T1 in the study group (*E* = 17.71, SE = 4.44, 95% CI [8.76, 26.66], *t*(44) = 3.99, *p* < 0.001), whereas no statistically significant change was observed in the control group (*E* = −2.72, SE = 4.44, 95% CI [−11.67, 6.22], *t*(44) = −0.61, *p* = 0.542) (Figure 2). All pairwise post-hoc contrasts for SDNN are reported in Supplementary Table S3.

The analysis revealed no statistically significant main effect of time in RMSSD scores, *F*(1, 44) = 3.06, *p* = 0.087, *f* = 0.26, and no statistically significant main effect of group, *F*(1, 42) = 1.36, *p* = 0.251, *f* = 0.18. No statistically significant time × group interaction was observed, *F*(1, 44) = 2.16, *p* = 0.149, *f* = 0.22, indicating that scores over time did not significantly differ between groups. Among the covariates, age was statistically significant, *F*(1, 42) = 10.67, *p* = 0.002, *f* = 0.50, while gender was not statistically significant, *F*(1, 42) = 2.73, *p* = 0.106, *f* = 0.25.

### 3.4 Pulmonary function

The analysis revealed no statistically significant main effect of time in FEV1 scores, *F*(1, 43) = 3.22, *p* = 0.080, *f* = 0.27, while the main effect of group was statistically significant, *F*(1, 41) = 4.07, *p* = 0.050, *f* = 0.31. No statistically significant time × group interaction was observed, *F*(1, 43) = 0.04, *p* = 0.849, *f* = 0.03, indicating that scores over time did not significantly differ between groups. Among the covariates, age was statistically significant, *F*(1, 41) = 15.67, *p* < 0.001, *f* = 0.62, as well as gender, *F*(1, 41) = 58.79, *p* < 0.001, *f* = 1.20.

The analysis revealed no statistically significant main effect of time in FVC, *F*(1, 43) = 0.05, *p* = 0.819, *f* = 0.04, while a statistically significant main effect of group was found, *F*(1, 41) = 6.80, *p* = 0.013, *f* = 0.41. No statistically significant time × group interaction was observed, *F*(1, 43) = 0.32, *p* = 0.575, *f* = 0.09, indicating that scores over time did not significantly differ between groups. Among the covariates, age was statistically significant, *F*(1, 41) = 7.86, *p* = 0.008, *f* = 0.44, as well as gender, *F*(1, 41) = 61.79, *p* < 0.001, *f* = 1.23.

The analysis revealed a statistically significant main effect of time in FEV1/FVC, *F*(1, 43) = 4.91, *p* = 0.032, *f* = 0.34, whereas the main effect of group was not statistically significant, *F*(1, 41) = 1.46, *p* = 0.233, *f* = 0.19. The time × group interaction was not statistically significant, *F*(1, 43) = 1.15, *p* = 0.289, *f* = 0.16, indicating that changes over time did not significantly differ between groups. Among the covariates, age was statistically significant, *F*(1, 41) = 5.43, *p* = 0.025, *f* = 0.36, while gender was not statistically significant, *F*(1, 41) = 0.38, *p* = 0.542, *f* = 0.10.

### 3.5 Muscle flexibility

The analysis revealed no statistically significant main effect of time in Sit and reach scores, *F*(1, 44) = 0.003, *p* = 0.960, *f* = 0.01, and no statistically significant main effect of group, *F*(1, 42) = 0.32, *p* = 0.577, *f* = 0.09. The time × group interaction was not statistically significant, *F*(1, 44) = 2.14, *p* = 0.151, *f* = 0.22, indicating that scores over time did not significantly differ between groups. Among the covariates, age was not statistically significant, *F*(1, 42) = 0.21, *p* = 0.649, *f* = 0.07, whereas gender was statistically significant, *F*(1, 42) = 10.53, *p* = 0.002, *f* = 0.50.

## 4 Discussion

The current study aimed to explore the effects of an 8-week mindfulness-based training in a group of athletes on several performance-relevant psychological and physiological measures, compared to a control group that did not participate in the training. The results revealed significant improvements on some of these variables in athletes, providing guidance about the potential benefits of mindfulness in the sports field.

Specifically, the results from MAIA showed a statistically significant improvement in the study group compared to the control group following treatment. This indicates that mindfulness-based training improved participants’ interoceptive awareness. The result is in line with literature indicating that a brief mindfulness training can promote the ability to consciously perceive and integrate signals from the body (Lima-Araujo et al., 2022), by cultivating present-moment focused attention to internal experience, such as breath and bodily sensations (Kabat-Zinn, 2003; Lutz et al., 2008; Creswell et al., 2019; Tang et al., 2015). This observed enhancement could provide different potential benefits for athletes. First, it could lead to better motor control or coordination (Bruscolotti et al., 2020; Li et al., 2021). Indeed, athletes who are more attuned to their internal cues could be able to fine-tune their movements more effectively. This is particularly relevant in high-level sports, where precise motor execution is critical for performance optimization. Greater sensitivity to physiological cues such as muscle response, respiration and heart rate, or fatigue signals, could also allow athletes to adjust exercise intensity in real time based on these cues during both training and competition. In case of fatigue signals during or after exercise, being aware of early signs of exhaustion could enable proactive recovery strategies. This kind of self-regulation could help reduce the risk of over-exertion-related injuries (Bruscolotti et al., 2020; Gledhill, 2021). Overall, the result of the questionnaire confirms the extensive literature supporting mindfulness as a long-standing contemplative practice for expanding awareness and being more aware of self and internal dynamics (Kabat-Zinn, 2003). In relation to the cognitive dimension, the results from Stroop task showed no statistically significant improvement in the study group compared to the control group following treatment on Interference index. This finding is in contrast with the existing literature, as several studies have reported improvements following an 8-week mindfulness training in attentional (Sumantry and Stewart, 2021; Verhaeghen, 2021; Chiesa et al., 2011) and executive functions, particularly in cognitive inhibition (Sumantry and Stewart, 2021; Verhaeghen, 2021; Cásedas et al., 2020). These cognitive domains would be employed during the meditative practice and appear to improve following the practice, as also does the activity of the brain areas that mediate these processes (Tang et al., 2015). However, our study did not find improvements in a test measuring these domains, which remain relevant to athletic performance in many disciplines (Brimmell et al., 2024; Moran, 2012).

The results from HRV showed a statistically significant increase in the study group compared to the control group following treatment in the SDNN parameter. This finding is consistent with previous studies analyzing the effects of mindfulness on HRV with other populations (Tung and Hsieh, 2019; Heckenberg et al., 2018). In particular, the SDNN index reflects the balance between the sympathetic and parasympathetic systems (Sztajzel, 2004). Thus, this improvement suggests that mindfulness-based training positively affects the athletes’ overall balance of the ANS, potentially contributing to improved stress adaptability, mental and physical health (Kemp and Quintana, 2013), and cardiovascular function (Xhyheri et al., 2012). These benefits are particularly valuable for athletes, as enhanced autonomic balance and stress regulation can directly impact both performance and recovery (Plews et al., 2013). A more balanced autonomic nervous system supports a quicker return to homeostasis following intense physical exertion, reducing recovery times, and lowering the risk of overtraining and injury (Plews et al., 2013). Moreover, cardiovascular function is crucial in most sports. For example, in sports requiring sustained aerobic effort or repeated high-intensity workouts—such as running, cycling, soccer, or basketball—optimal cardiovascular efficiency becomes a key determinant of endurance and overall physical performance. An efficient cardiovascular function ensures that working muscles receive an adequate supply of oxygen and nutrients during exercise, while also facilitating the removal of metabolic by-products (Mazaheri et al., 2021; Joyner and Coyle, 2008). This can delay the onset of fatigue and improve recovery capacity between training sessions and competitive events. Improved HRV indices have also been associated with psychological wellbeing, positive emotionality, emotional regulation, and motivation (Kemp and Quintana, 2013), all crucial skills for athletes competing at high levels (Schinke et al., 2018). Therefore, the observed benefit could be translated into both physiological and psychological advantages for athletes, helping them sustain high levels of performance while maintaining psychophysical wellbeing. Regarding the RMSSD parameter—which primarily reflects parasympathetic activity (Sztajzel, 2004)—the results showed no statistically significant improvement in the study group compared to the control group following treatment on this parameter, in contrast with previous research (Barbry et al., 2025; Tung and Hsieh, 2019). This pattern may suggest that, at this early stage of practice and in a population of athletes—who should already be relatively healthy from a cardiovascular perspective—mindfulness primarily influences overall autonomic balance rather than producing a pronounced increase in parasympathetic tone. Additionally, the results showed a statistically significant effect of age on both SDNN and RMSSD parameters. This indicates that an increase in age corresponds to lower values of these indices, reflecting a gradual decline in overall autonomic flexibility. This pattern is in line with the previous literature (Shaffer and Ginsberg, 2017), as HRV tends to decrease across the lifespan due to age-related changes in cardiac autonomic regulation.

Concerning the pulmonary function, the results showed no improvement in the study group compared to the control group following treatment on FEV1, FVC, and FEV1/FVC parameters. Therefore, our results did not suggest short-term benefits on pulmonary function in athletes due to brief mindfulness training and its breathing techniques. Beyond the rapid and irregular breathing rates linked to sympathetic hyperactivation, anxiety and stress conditions (Jerath et al., 2015), the available evidence on this topic was limited and focused upon changes on respiratory rate in healthy meditators (Karunarathne et al., 2024), and on psychological outcomes and airway inflammation biomarkers in meditators with respiratory disorders (Arefnasab et al., 2013; Paudyal et al., 2018; Li et al., 2023). In these populations, no improvements following mindfulness have been reported so far on these pulmonary function parameters (Karunarathne et al., 2024; Arefnasab et al., 2013; Paudyal et al., 2018). Our result is in line with this previous research. Moreover, age showed a statistically significant effect on FEV1, FVC and FEV1/FVC parameters, suggesting that a an increase in age corresponds to lower values on these indices, while gender showed a statistically significant effect on FEV1 and FVC, suggesting that males exhibited higher values on these two indices. Both results are in accordance with previous research indicating that the spirometry values tend to progressively decrease with advancing age, and that males have higher FEV1 and FVC—but not FEV1/FVC—values than females due to largest height and chest size (Stanojevic et al., 2008).

Similarly, the results of Sit and reach test showed no improvement in the study group compared to the control group following treatment. Therefore, our results did not suggest short-term benefits on muscle flexibility in athletes due to brief mindfulness training and its relaxation techniques. On a theoretical level, mindfulness has been proposed to facilitate psychophysical relaxation (Kabat-Zinn, 2003; Luberto et al., 2020) and has demonstrated efficacy in reducing stress-related arousal (Tung and Hsieh, 2019; Heckenberg et al., 2018; Keng et al., 2011), which may in turn contribute to the reduction of dysfunctional muscle tension (Pluess et al., 2009). This may, over time, facilitate improvements in flexibility. Indeed, excessive and maladaptive muscle tension is known to limit joint range of motion, a process that negatively affects athletic performance and increases injury risk (Olmedilla-Zafra et al., 2017; Witvrouw et al., 2003; Williams and Andersen, 1998). However, so far, while some studies suggest positive effects of mindfulness-based interventions on musculoskeletal disorders symptoms (Hilton et al., 2017; Cruze and Games, 2021), and a decrease in the electrical activity of muscle fibers (Crescentini et al., 2016), no further data are available on muscle-related outcomes in healthy and athletic populations. Our finding adds to this limited body of evidence. Moreover, a statistically significant effect of gender was showed on the scores of this test, indicating that females exhibit a greater score than males. This is another result in line with the previous literature showing that females practicing different sports had higher values of muscle flexibility measured with this test than males (Aedo-Muñoz et al., 2019). Finally, it is worth noting that, although the analysis revealed descriptive improvements in some outcomes (e.g., FEV1/FVC and muscle flexibility) in the study group compared to the control group following the treatment, these changes were not statistically significant. This lack of significance may be partially attributed to the limited statistical power to detect smaller effects. As indicated by the *post hoc* sensitivity power analysis, the sample size was sufficient to detect only medium-to-large effects (Cohen, 2013). Therefore, it is plausible that smaller effects, potentially present in these outcomes, remained undetected due to sample size constraints.

In conclusion, this study provides preliminary evidence supporting the positive effects of mindfulness-based training in athletes on interoceptive awareness and SDNN parameter of HRV. Notably, this study offers an integrated mind–body approach to the efficacy of mindfulness, simultaneously assessing psychological and physiological outcomes that could be translated into sports context. However, further research is needed to confirm and extend these effects. A deeper understanding of the mechanisms underlying mindfulness practice could provide a solid scientific basis for promoting the integration of this practice into sports training programs. This would offer a more holistic and ecological approach to optimizing the mental and physical balance of athletes, but also to provide applications into clinical and non-clinical contexts for the general wellbeing of people.

## 5 Limitations and future directions

The results of our study provide preliminary indications and should be interpreted with caution, due to several limitations. First, the study sample size was relatively small (<50 participants), and most of the athletes practiced CrossFit. This limits the generalization of the results and the extensibility to the entire population of athletes. Furthermore, our study focused on a relatively short treatment period (only 8 weeks), which may limit the manifestation of the stable effects of the practice. Therefore, future research should conduct longer-duration longitudinal studies with larger and more heterogeneous samples of athletes to confirm and expand our evidence and evaluate the long-term effects of mindfulness. For instance, such studies may determine whether the non-significant descriptive improvements observed here persist or strengthen and reflect small effects detectable under conditions of increased statistical power. Another limitation concerns the use of the MAIA questionnaire: although this instrument includes eight distinct subscales assessing different dimensions of interoceptive awareness, in the present study a single composite score was calculated instead of separate subscale scores. This approach may have reduced the specificity of the findings regarding the various components of interoceptive awareness. Direct measures of sport performance are also needed to clarify whether and how the observed changes translate into performance-related outcomes. Finally, a further limitation of our study is that the training method did not consist of a standardized protocol but applied two of the most widely used techniques in mindfulness-based interventions. It may reduce reproducibility and make it more difficult to compare our findings with studies that adopt a standardized program.

## Data Availability

The raw data supporting the conclusions of this article will be made available by the authors without undue reservation.
