# Supplementary material for: Mind–body effects of mindfulness-based training in athletes: a preliminary randomized controlled trial
Source: Front Psychol. 2026 Feb 19;17:1755043. doi: 10.3389/fpsyg.2026.1755043 (PMC12978131; doi:10.3389/fpsyg.2026.1755043)
Supplement: Supplementary file 1 [file Table_1.docx]

Supplementary Material

# Supplementary Tables

|  |  | | | **95% Confidence Intervals** | |  | | |
| --- | --- | --- | --- | --- | --- | --- | --- | --- |
|  | **Parameter** | **Estimate** | **SE** | **Lower** | **Upper** | **df** | **t** | **p** |
| MAIA | (Intercept) | 2.87 | 0.09 | 2.67 | 3.05 | 42 | 30.40 | <0.001 |
|  | Time | 0.22 | 0.08 | 0.06 | 0.37 | 44 | 2.79 | 0.008 |
|  | Group | 0.04 | 0.19 | -0.33 | 0.42 | 42 | 0.22 | 0.830 |
|  | Age | 0.02 | 0.01 | 0.00 | 0.04 | 42 | 1.91 | 0.063 |
|  | Gender | -0.10 | 0.20 | -0.50 | 0.31 | 42 | -0.48 | 0.632 |
|  | Group ✻ Time | 0.36 | 0.15 | 0.06 | 0.67 | 44 | 2.36 | 0.023 |
| Interference | (Intercept) | 276.44 | 32.78 | 211.16 | 341.72 | 38 | 8.43 | <0.001 |
|  | Time | -88.37 | 43.55 | -175.11 | -1.62 | 40 | -2.03 | 0.049 |
|  | Group | 66.25 | 66.01 | -65.23 | 197.73 | 38 | 1.00 | 0.322 |
|  | Age | 5.51 | 3.74 | -1.93 | 12.95 | 38 | 1.48 | 0.148 |
|  | Gender | -131.22 | 70.18 | -271.00 | 8.56 | 38 | -1.87 | 0.069 |
|  | Group ✻ Time | 119.91 | 87.11 | -53.58 | 293.40 | 40 | 1.38 | 0.176 |
| SDNN | (Intercept) | 66.12 | 4.02 | 58.13 | 74.10 | 42 | 16.47 | <0.001 |
|  | Time | 7.49 | 3.14 | 1.25 | 13.74 | 44 | 2.39 | 0.021 |
|  | Group | 10.32 | 8.05 | -5.69 | 26.33 | 42 | 1.28 | 0.207 |
|  | Age | -1.75 | 0.42 | -2.60 | -0.91 | 42 | -4.14 | <.001 |
|  | Gender | -8.69 | 8.68 | -25.96 | 8.57 | 42 | -1.00 | 0.322 |
|  | Group ✻ Time | 20.44 | 6.28 | 7.95 | 32.93 | 44 | 3.25 | 0.002 |
| RMSSD | (Intercept) | 57.87 | 4.51 | 48.90 | 66.84 | 42 | 12.82 | <0.001 |
|  | Time | 6.78 | 3.88 | -0.93 | 14.49 | 44 | 1.75 | 0.087 |
|  | Group | 10.53 | 9.05 | -7.46 | 28.52 | 42 | 1.16 | 0.251 |
|  | Age | -1.56 | 0.48 | -2.51 | -0.61 | 42 | -3.27 | 0.002 |
|  | Gender | -16.13 | 9.76 | -35.54 | 3.27 | 42 | -1.65 | 0.106 |
|  | Group ✻ Time | 11.39 | 7.75 | -4.02 | 26.81 | 44 | 1.47 | 0.149 |
| FVC | (Intercept) | 4.99 | 0.10 | 4.79 | 5.19 | 41 | 50.61 | <0.001 |
|  | Time | 0.01 | 0.04 | -0.07 | 0.09 | 43 | 0.23 | 0.819 |
|  | Group | -0.52 | 0.20 | -0.91 | -0.12 | 41 | -2.61 | 0.013 |
|  | Age | -0.03 | 0.01 | -0.05 | -0.01 | 41 | -2.80 | 0.008 |
|  | Gender | 1.66 | 0.21 | 1.24 | 2.08 | 41 | 7.86 | <0.001 |
|  | Group ✻ Time | -0.04 | 0.08 | -0.20 | 0.11 | 43 | -0.57 | 0.575 |
| FEV1 | (Intercept) | 4.15 | 0.08 | 3.99 | 4.31 | 41 | 51.10 | <.001 |
|  | Time | 0.07 | 0.04 | -0.01 | 0.14 | 43 | 1.79 | 0.080 |
|  | Group | -0.33 | 0.16 | -0.65 | 0.00 | 41 | -2.02 | 0.050 |
|  | Age | -0.03 | 0.01 | -0.05 | -0.02 | 41 | -3.96 | <0.001 |
|  | Gender | 1.34 | 0.17 | 0.99 | 1.68 | 41 | 7.67 | <0.001 |
|  | Group ✻ Time | 0.01 | 0.07 | -0.13 | 0.16 | 43 | 0.19 | 0.849 |
| FEV1/FVC | (Intercept) | 83.37 | 0.75 | 81.88 | 84.87 | 41 | 110.86 | <0.001 |
|  | Time | 1.28 | 0.58 | 0.13 | 2.42 | 43 | 2.22 | 0.032 |
|  | Group | 1.82 | 1.51 | -1.18 | 4.82 | 41 | 1.21 | 0.233 |
|  | Age | -0.19 | 0.08 | -0.34 | -0.03 | 41 | -2.33 | 0.025 |
|  | Gender | -0.99 | 1.61 | -4.20 | 2.22 | 41 | -0.62 | 0.542 |
|  | Group ✻ Time | 1.24 | 1.15 | -1.06 | 3.53 | 43 | 1.07 | 0.289 |
| Sit and reach | (Intercept) | 7.97 | 1.13 | 5.73 | 10.21 | 42 | 7.08 | <0.001 |
|  | Time | 0.01 | 0.29 | -0.56 | 0.59 | 44 | 0.05 | 0.960 |
|  | Group | -1.27 | 2.26 | -5.76 | 3.22 | 42 | -0.56 | 0.577 |
|  | Age | 0.05 | 0.12 | -0.18 | 0.29 | 42 | 0.46 | 0.649 |
|  | Gender | -7.90 | 2.44 | -12.75 | -3.06 | 42 | -3.24 | 0.002 |
|  | Group ✻ Time | 0.84 | 0.58 | -0.30 | 1.98 | 44 | 1.46 | 0.151 |

***Table S1.*** *Parameter estimates of linear mixed-effects models for each outcome.*

| **Comparison** | | | | |  | | **95% Confidence Intervals** | |  | | |
| --- | --- | --- | --- | --- | --- | --- | --- | --- | --- | --- | --- |
| **Group** | **Time** | **vs** | **Group** | **Time** | **Difference** | **SE** | **Lower** | **Upper** | **t** | **df** | **p** |
| Mindfulness | T0 | - | Mindfulness | T1 | -0.40 | 0.11 | -0.61 | -0.18 | -3.64 | 44.0 | <.001 |
| Mindfulness | T0 | - | Control | T0 | -0.14 | 0.20 | -0.55 | 0.27 | -0.69 | 55.7 | 0.492 |
| Mindfulness | T0 | - | Control | T1 | -0.17 | 0.20 | -0.58 | 0.23 | -0.86 | 55.7 | 0.396 |
| Mindfulness | T1 | - | Control | T0 | 0.26 | 0.20 | -0.15 | 0.66 | 1.26 | 55.7 | 0.215 |
| Mindfulness | T1 | - | Control | T1 | 0.22 | 0.20 | -0.19 | 0.63 | 1.09 | 55.7 | 0.280 |
| Control | T0 | - | Control | T1 | -0.03 | 0.11 | -0.25 | 0.19 | -0.31 | 44.0 | 0.761 |

***Table S2.*** *Pairwise post-hoc contrasts for MAIA.*

| **Comparison** | | | | |  | | **95% Confidence Intervals** | |  | | |
| --- | --- | --- | --- | --- | --- | --- | --- | --- | --- | --- | --- |
| **Group** | **Time** | **vs** | **Group** | **Time** | **Difference** | **SE** | **Lower** | **Upper** | **t** | **df** | **p** |
| Mindfulness | T0 | - | Mindfulness | T1 | -17.71 | 4.44 | -26.67 | -8.76 | -3.99 | 44.0 | <.001 |
| Mindfulness | T0 | - | Control | T0 | 0.10 | 8.64 | -17.22 | 17.42 | 0.01 | 54.5 | 0.991 |
| Mindfulness | T0 | - | Control | T1 | 2.83 | 8.64 | -14.50 | 20.15 | 0.33 | 54.5 | 0.745 |
| Mindfulness | T1 | - | Control | T0 | 17.81 | 8.64 | 0.49 | 35.14 | 2.06 | 54.5 | 0.044 |
| Mindfulness | T1 | - | Control | T1 | 20.54 | 8.64 | 3.22 | 37.86 | 2.38 | 54.5 | 0.021 |
| Control | T0 | - | Control | T1 | 2.73 | 4.44 | -6.22 | 11.68 | 0.61 | 44.0 | 0.542 |

***Table S3.*** *Pairwise post-hoc contrasts for SDNN.*
